# Proteomic Approaches for the Discovery of Biofluid Biomarkers of Neurodegenerative Dementias

**DOI:** 10.3390/proteomes6030032

**Published:** 2018-08-31

**Authors:** Becky C. Carlyle, Bianca A. Trombetta, Steven E. Arnold

**Affiliations:** Massachusetts General Hospital Department of Neurology, Charlestown, MA 02129, USA; bcarlyle@mgh.harvard.edu (B.C.C.); btrombetta@partners.org (B.A.T.)

**Keywords:** neurodegeneration, Alzheimer’s disease, cerebrospinal fluid, plasma, serum, proteomics, biomarkers, LC-MS/MS

## Abstract

Neurodegenerative dementias are highly complex disorders driven by vicious cycles of intersecting pathophysiologies. While most can be definitively diagnosed by the presence of disease-specific pathology in the brain at postmortem examination, clinical disease presentations often involve substantially overlapping cognitive, behavioral, and functional impairment profiles that hamper accurate diagnosis of the specific disease. As global demographics shift towards an aging population in developed countries, clinicians need more sensitive and specific diagnostic tools to appropriately diagnose, monitor, and treat neurodegenerative conditions. This review is intended as an overview of how modern proteomic techniques (liquid chromatography mass spectrometry (LC-MS/MS) and advanced capture-based technologies) may contribute to the discovery and establishment of better biofluid biomarkers for neurodegenerative disease, and the limitations of these techniques. The review highlights some of the more interesting technical innovations and common themes in the field but is not intended to be an exhaustive systematic review of studies to date. Finally, we discuss clear reporting principles that should be integrated into all studies going forward to ensure data is presented in sufficient detail to allow meaningful comparisons across studies.

## 1. Introduction

Clinical neuroscientists and practitioners have gained access to an increasing array of tools to assist in the diagnosis of neurodegenerative disease dementias. Various neuroimaging techniques and a number of cerebrospinal fluid (CSF) biomarkers can now complement diagnosis that was once based solely on careful clinical and neuropsychological assessments of symptoms and only positively confirmed at autopsy [1]. These additional biomarkers can be extremely informative, as many neurological diseases present with similar sets of cognitive, behavioral, and/or movement symptoms, particularly in early disease stages. While neuroimaging-based techniques, including structural and functional Magnetic Resonance Imaging (MRI) and Positron Emission Tomography (PET), are currently the most commonly used diagnostic measures, these require sophisticated on-site technologies and expertise in specialized centers and they are expensive [2]. The field could benefit from increasing availability of biomarkers in blood, CSF, or other biofluids, which are more widely attainable through minimally invasive means, simpler to interpret, and performed on more routine diagnostic equipment [3].

A series of National Institute on Aging and Alzheimer Association consensus conferences suggested a number of criteria that a biomarker of neurodegenerative disease should fulfill [4]. A putative marker should be linked to the fundamental neuropathology of the disease and validated in neuropathologically confirmed cases. Ideally, a marker would be able to detect the disease before the onset of symptoms, distinguish between neurodegenerative disorders, and not be affected by treatment with symptom-relieving drugs. Practically, a marker should be non- or minimally invasive, simple to execute, and relatively inexpensive. Based on these principles, a new research framework, “AT(N)”, was proposed for clear delineation of Alzheimer’s disease (AD) from other disorders. In this framework [1], an indication of amyloid pathology (A+) by amyloid PET or in CSF is necessary for assigning a subject to an AD diagnosis. The disease can be further classified by the presence or absence of tau fibrillation (T), measured by PET or phosphorylated-tau (pTau) in CSF, and the extent of neurodegeneration (N) as measured by structural MRI or total tau in CSF. Despite this improvement in defining AD in biological terms, these markers alone do not allow for clear staging and AD prognosis. For example, the definition of a case as A+T+ may predict progression of a subject from mild cognitive impairment (MCI) to dementia but with a highly variable timeframe. As a result of this variability, the AT(N) framework was designed to flexibly accommodate the addition of further biomarker groups such as vascular and synuclein markers that may aid in the overall characterization of neurodegenerative disorders as distinct clinical entities and likely treatment groups.

Biofluids fulfill the practicality recommendations for a biomarker, being relatively easily and economically attainable. CSF is the primary fluid of choice, being in intimate contact with the interstitial fluid of the brain and carrying molecules secreted by neurons and glia, excreted metabolic waste, and material from dying synapses, axons, and cells that indicate neurodegeneration [5,6,7]. However, although the lumbar puncture procedure to obtain CSF is generally considered straightforward, safe, and tolerable, it is not routinely performed in many neurology clinics due to patient and clinician disinclination [8,9]. The procedure is also not particularly well suited to multiple short-term repeat measures, such as those used to assess target engagement, pharmacokinetics, or acute pharmacodynamic response of a novel drug. This had led to a widespread belief that the “holy grail” of neurodegenerative disease research lies in a blood-based biomarker [10]. 

In blood-derived fluids (plasma and serum), central nervous system (CNS)-specific proteins are diluted by proteins from all other peripheral tissue sources, leading to potentially low concentrations that require ultrasensitive quantification [6,7]. Proteins may be regulated and modified by different processes in the CNS versus the periphery, resulting in a lack of correlation between abundance in CSF and blood [11,12]. Blood may also be presumed to be more labile, being in contact with many more secretory and excretory tissues than CSF. Finally, blood, and to a lesser extent CSF, is a complex mixture of proteins and metabolites that span a large range of abundances. In plasma, protein concentrations range from the most abundant protein, human serum albumin at 50 mg/mL, to signaling proteins in the low pg/mL range, such as IL-6 [13,14,15]. These large differences in protein abundance mean there is currently no perfect technique for quantifying a large number of analytes that span this dynamic range.

**Figure 1:**
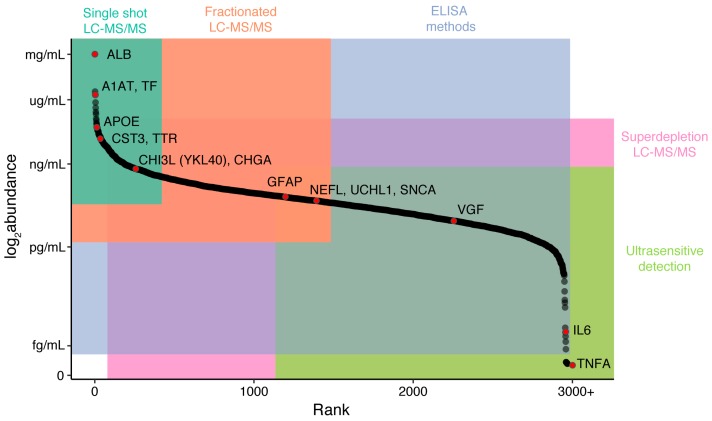
Different proteomic techniques are more suited to different concentration ranges of biofluid analytes. In this plot, cerebrospinal fluid (CSF) proteins are ranked according to their abundance, with the location of specific proteins placed according to their concentrations in enzyme-linked immunoassays (ELISAs), Multiple-Reaction-Monitoring (MRM), and in-house (unpublished) label-free experiments [23,24,25,26]. It is of note that there is a large amount of disagreement between experiments on the exact concentrations of these analytes, and so their place on this plot should be considered illustrative. Of particular note is VGF, an analyte that exists as multiple processed peptides, which is easily detected by single-shot LC-MS/MS but detected in the low pg/mL ranges by ELISA. Single-shot LC-MS/MS will generally quantify 300–500 abundant proteins in CSF (turquoise), and protein identifications can be increased by offline fractionation of samples (orange). While ELISA-based methods measure analytes across the widest concentration range, these techniques require a strong hypothesis for target selection and rely on the availability of an appropriate antibody pair for the analyte. At low analyte concentrations, super depletion can be combined with LC-MS/MS to reveal low-abundance proteins, but there are concerns over nonspecific depletion of some target analytes. Finally, ultrasensitive platforms can be used to measure proteins such as cytokines in CSF, which are present in the low pg/mL to fg/mL range.

Proteomic approaches are an excellent companion in the search for novel neurodegenerative disease biomarkers. Recent improvements in reproducibility and sensitivity of liquid chromatography tandem mass spectrometry (LC-MS/MS) instrumentation [16], coupled with the development of immunoassay-based single molecule quantification and multiplexing [17,18,19,20,21,22], offer a wide range of tools to allow for hypothesis-free target discovery through to the ability to accurately, sensitively, and simultaneously quantify a specific small number of targets. While proteomic techniques are available that together span most of the range of protein abundances in a complex biofluid, from ultrasensitive (~0.05 pg/mL) through to extremely abundant (~50 mg/mL), careful experimental selection and design is important to maximize the likelihood of accurately quantifying a target of interest (Figure 1). In this review, we introduce a toolbox of techniques available to the biomarker researcher, the advantages and disadvantages of the major technologies, and finally, some of the key discoveries to date in the field of protein biomarkers for neurodegeneration.

## 2. LC-MS/MS Strategies

Most basic LC-MS/MS proteomic workflows derive from the same underlying tandem mass-spectrometry method [27]. A protease-digested peptide mixture is injected onto a liquid chromatography column, then eluted from the column with a solvent gradient over a period of time. Peptides enter the tandem mass spectrometer, where they are ionized (“precursor ion”), separated by mass charge ratio, and detected. In data-dependent methods, the first “MS1” detection is generally used to quantify the peptides. In most workflows, a subset of precursor ions is isolated and fragmented (“fragment ions”) for a second round of mass spectrometry (MS2). MS2 fragments can be used for both confident identification of a peptide and for peptide quantification [28]. Almost every step of this simple workflow, including sample preparation, can be tweaked to optimize the parameters of the experiment, providing an extremely flexible basic platform for biomarker discovery across a range of analyte concentrations [29,30,31].

### 2.1. Data-Dependent LC-MS/MS

Label-free methods are the simplest LC-MS/MS workflows. In these experiments, an unlabeled peptide sample is injected directly onto the instrument-coupled LC column and quantified by MS1 intensity or spectral counting [32,33,34,35]. Peptides are identified by matching of the MS2 fragmentation products to the spectral properties of known peptides in a database. As only a single “snapshot” MS2 measurement is taken, accurate MS2 level quantification is not possible. Each sample is injected independently, and experimental reproducibility is highest if these injections are performed consecutively with careful monitoring of LC performance [36,37]. For this reason, it may be difficult to directly compare quantification from two label-free experiments carried out at different times in different labs or with a different LC setup.

While this method enables truly hypothesis-free biomarker discovery without the need for antibodies, there are a number of disadvantages to using label-free techniques that are of particular importance in biofluids. The greatest disadvantage is that peptides from high-abundance proteins such as albumin can mask or interfere with peptides from lower-abundance proteins, decreasing the sensitivity of the experiment [38,39]. While it is possible to simplify the peptide mixture entering the instrument by increasing the length of the elution from the LC, the number of protein identifications in brain tissue currently tends to plateau at between 3000 and 5000 proteins [40]. In biofluids such as blood, where albumin and the immunoglobulins make up more than 75% of total protein weight, and a further 20 proteins account for more than 24% of the total weight, this masking is profound. A standard long-gradient (>2.5 h) label-free experiment in blood yields identification of approximately 300 of the most abundant proteins [13], which may not be sufficiently sensitive (Figure 1).

Two main approaches have been used to increase the sensitivity of data-dependent approaches. In the first, samples are prefractionated offline, simplifying the injection mixture and spreading out spectra to decrease the impact of peptide masking from abundant peptides [30]. In unlabeled experiments, this can lead to quantification difficulties, as normalizing across multiple injections is complex. To get around this issue, individual samples can be labeled using a sample-specific isobaric tag (TMT or iTRAQ) [41,42,43]. Tagging results in coelution of isobaric precursors from all multiplexed samples that can then be assigned to individual samples at the MS2 fragment stage. Peptides are quantified at the MS2 level, and a relative abundance is obtained for each peptide in each sample, removing the need to normalize across injections. While the sensitivity of this technique to small fold changes is high, large fold changes may be compressed [44,45,46]. This approach improves the overall depth of the experiment to an extent determined by the number of offline fractions run [42,47] but is not always sensitive enough to detect proteins only found in a small number of the multiplexed samples. In their proof-of-principle paper, Russell et al. [48] leveraged this potential weakness by combining CSF samples with microglial cell line (BV2) lysate samples to improve detection of immune related proteins, which are low abundance and generally difficult to detect by LC-MS/MS in CSF. Presence of strong MS1 spectra driven by the BV2 cell calibrator drives data-dependent MS2 level acquisition, allowing for quantification of peptides that would not normally be acquired in CSF samples alone. Forty-one proteins that had not previously been identified in CSF were found to differ in abundance between AD and control subjects. The utility of this approach to drive acquisition of data from low-abundance CNS-derived proteins in plasma should be tested. 

The second approach to increasing the sensitivity of data-dependent experiments is to deplete samples of the most abundant proteins to decrease interference from these proteins. The standard approach is immunodepletion, using immobilized antibodies to remove abundant proteins from the biofluid sample. While this technique does increase the sensitivity to a subset of lower abundance proteins, nonspecific interactions between the immunodepletion matrix and specific protein–protein interactions between the depletion targets and other proteins can lead to off-target depletion of proteins [49,50]. Therefore, it is important to run pilot experiments or search publicly available data to assess the effect that immunodepletion may have on particular proteins of interest. In plasma, where the dominance by abundant proteins is more extreme, Keshisian et al. [51] reported using a super depletion technique (of approximately 60 of the most abundant proteins) that was combined with isobaric labeling and offline fractionation to confidently identify over 5000 proteins in plasma samples, highlighting several novel candidates for detecting early myocardial infarction. While these approaches may prove useful in discovery experiments, it is likely that such a procedure would introduce much variation and be too costly for routine clinical or large-scale research use.

### 2.2. Targeted LC-MS/MS Acquisition

If an investigator already has an analytes(s) of interest, then a targeted approach such as selected reaction monitoring (SRM) [52,53] or parallel/multiple reaction monitoring (PRM/MRM) [54,55,56] may be the preferred approach. These methods quantify at the MS2 level, allowing for better precision and more accurate peptide quantification than data-dependent methods [57]. From a user perspective, the main difference between SRM and PRM is the number of peptides that can be quantified [58]. In SRM, each precursor-fragment pair (“transition”) must be independently scanned for quantification, whereas in PRM, all fragments from the same precursor are simultaneously scanned, allowing quantification of a greater number of targets. Work-up time is also therefore shorter for PRM, as individual transitions do not need to be manually selected [59,60]. Scheduling (looking for a precursor only at a specific retention time range) can increase the number of targets included in either method but may lead to missing data in cases where there is significant LC drift. In both methods, it is best to use data-dependent acquired libraries generated on the same LC setup and instrument that the targeted methods will be performed on to begin the precursor and fragment selection process. Due to the lower number of targets quantified, targeted experiments, particularly those using SRM, are often performed using heavy labeled standards, and as a result, are currently seen as the gold standard in LC/MS-MS quantification of proteins, lipids, and metabolites [54]. 

### 2.3. Data-Independent Acquisition

Data-independent acquisition (DIA/“Sequential Window Acquisition of All Theoretical Spectra” (SWATH)) sits at the intersection between data-dependent acquisition (DDA) and targeted approaches [61,62]. In a DIA method, acquisition is untargeted, with data acquired from tiled fragment scans that together span the whole mass/charge range. Each tile is repeated every instrument cycle, which allows for repeat measures and quantification for each MS2 fragment. Tiling of fragment scans results in a greater sensitivity than DDA approaches, allowing for higher throughput and shorter LC elution gradients. DIA is intermediate in accuracy between DDA and targeted methods and requires no advance work up [63,64]. Instead, data can be manually curated postacquisition, and removing poor quality fragments and peptides (such as those that exhibit interference from other ions) can vastly improve the precision of DIA, bringing it close to targeted methods. The sensitivity of DIA to lower abundance peptides was initially mostly dependent on the quality and depth of the libraries used to deconvolute MS2 data. These libraries can be generated on the instrument by preliminary DDA runs [65,66], but recently, there has been a proliferation in a number of tools that allow high-depth DIA analysis without the need for a comprehensive, user-generated peptide libraries (Spectronaut Pulsar, DIA Umpire [67], PeCan [68], EncyclopeDIA [69]). Scanning with variable size windows and overlapping tiles can also attain smaller but significant improvements in specificity and sensitivity [70,71]. A recent publication from Meier et al. [72] used DIA-like tiling approaches to replace the full m/z scan at the MS1 level, reducing suppression from abundant peptides and increasing ion injection time. Early data suggests this approach may greatly increase the depth of single-shot label-free techniques, allowing quantification of up to 10,000 proteins in an hour-long scan, with sensitivity down to attomolar levels. Fold change sensitivity and performance of this technique across large, multiday experiments is still to be established.

### 2.4. Candidate Disease Markers from LC-MS/MS Studies

**Figure 2:**
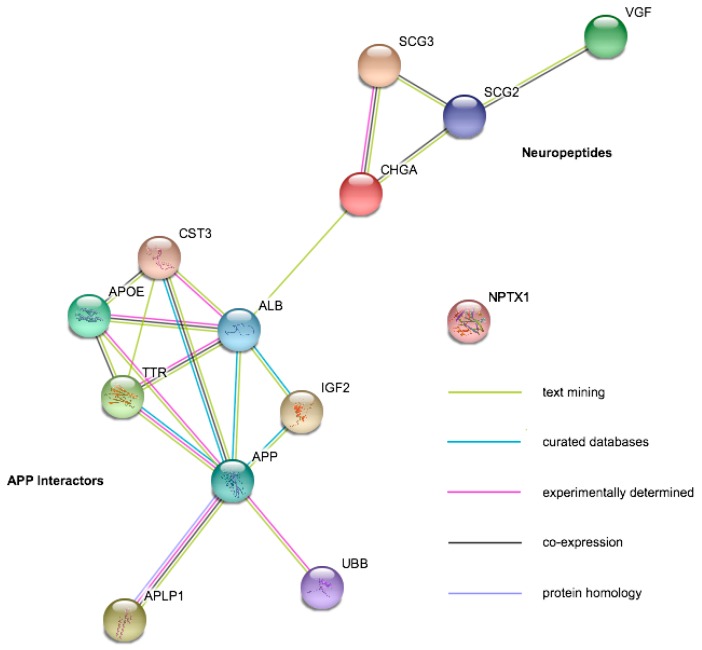
String [84] diagram shows functional protein relationships of proteins highlighted as potential CSF biomarkers of Alzheimer’s disease. These proteins currently fall into two main groups: neuropeptides and proteins that interact with amyloid precursor protein (APP, the precursor to beta-amyloid). The type of interaction can be determined from the key in the bottom right. Where peptides from the same protein differ in their significance, the reference is shown in more than one group.

Despite significant improvements in LC-MS/MS technology and an increasing adoption of these techniques, their utility thus far has been limited by low-powered studies, often utilizing pooling strategies that limit the assessment of individual heterogeneity of potential markers. The neurodegenerative disease biofluid biomarker field is currently dominated by studies of AD, with only a handful of studies on other conditions. In a review of LC-MS/MS studies performed in the last five years (see references [3,73] for comprehensive reviews of work prior to this), only a handful of potential targets were highlighted as significant between clinical groups by three or more studies in CSF, and there was no consensus from studies of blood. In plasma, there have been a number of hits in the complement factor cascade pathway but little agreement over which exact components may be dysregulated [74,75,76,77,78,79,80,81,82]. In CSF, potential targets fell into two main functional categories: neuropeptides (Chromogranin-A, Secretogranin-2, Secretogranin-3, Neurosecretory Protein VGF) and proteins that interact with amyloid precursor protein (APP) or its resulting peptides (Figure 2A). For all these proteins, there were studies that disagreed on the direction of change or that showed no abundance differences between AD and control (Table 1). There are also currently no markers that appear specific to a single neurodegenerative disease. The relatively low power of all of these studies (*n* per group ranging from 3 to 134 with a substantial right skew; it is also worth noting that the best powered study [83] found only one between-group difference that survived multiple testing correction) combined with differences in approach may account for a large amount of disagreement between studies. Targeted studies with fewer multiple tests are more likely to find significant outcomes, and correction is not always performed appropriately. Because original data is very rarely presented in these studies, it is difficult to re-examine data distributions, the effect of normalization, and assess whether a peptide was borderline significant or highly variable. In the Considerations for Accurate and Reproducible Findings section of this review, we discuss the adoption of minimum reporting standards to ensure improved reproducibility and comparability of future studies. 

A final reason for the discrepancies in this data may be that many proteins in biofluids exist not as intact peptides but as multiple processed peptides with differing functions, abundance, and stability [85,86,87]. The existence of these different proteoforms means that protein-level abundance values may vary wildly depending on which peptides are selected or detected in an assay. While targeted methods can be designed towards individual processed peptides to explicitly address this question, untargeted experiments quantified at the protein level only may produce confusing or conflicting results (Table 1). As understanding of the relationship of proteoforms to disease susceptibility increases, it is likely that there will be an expanding need for top-down proteomic methods, where intact peptides can be identified and quantified [88]. 

## 3. Capture-Based Strategies

Antibodies have long been the bedrock of protein quantification strategies, particularly in biofluid biomarker development. Antibodies are specific and flexible protein tools that can be easily conjugated to a number of different reporters and immobilized on a variety of matrices, allowing for their use in enzyme-linked immunoassays (ELISAs), Western blotting, and immunohistochemistry. Here, we focus on recent technological developments that allow for multiplexing of targets on ELISA-like platforms and ultrasensitive protein quantification, which may prove exceptionally useful in the detection of very low levels of CNS specific proteins in blood-derived biofluids. The reliance on antibodies for these techniques may result in problems, however [106]. The process of antibody production, particularly for polyclonal antibodies, can be subjected to large batch variation in antibody specificity. Antibody specificity can be difficult to test in human biofluids, where knockdown of a protein is not possible. Antibodies are commonly tested for cross-reactivity with spiked-in proteins that are structurally similar to the target, but nonspecificity can be difficult to predict and this approach is not exhaustive. It is therefore of critical importance to keep comprehensive documentation of lot numbers and batch numbers when performing antibody-based proteomic experiments to monitor potential unexpected causes of variation. 

### 3.1. Multiplexed Immunoassays

Although conventional colorimetric ELISA methods have remained the primary workhorse for measuring biomarker levels in biofluids, the emergence of electrochemiluminescent (ECL) immunoassay technology has allowed for the simultaneous measurement of multiple analytes across a broad dynamic range, leading ECL immunoassays to quickly become the new standard in the field [20,21]. ECL immunoassays are similar in workflow to traditional ELISAs. With plate-based immunoassays, such as those developed by MesoScale Discovery (MSD), carbon electrodes are coated with capture antibodies coated onto discrete spots in each plate well to allow multiplexing of up to 10 targets per sample. Secondary detection antibodies are conjugated to ECL labels that emit light when electricity is applied to the electrodes [21]. In contrast to ELISAs, which depend on developing colorimetric substrates over time, ECL immunoassays have heightened sensitivity with the application of multiple excitation cycles, which amplifies light intensity at lower levels and improves the signal-to-background ratio, enabling accurate measurements in the low pg/mL range [107]. Elimination of the chemical substrate also allows for more consistent and replicable detection, as ECL signal intensity does not vary over time. The increased sensitivity coupled with multiplexing capabilities allows for reduced sample volumes, lower per sample cost, and decreased processing time [108], which are critical considerations when working with valuable and limited biospecimens such as CSF.

Luminex Multi-Analyte Profiling (xMAP) technology uses color-coded beads bound to capture antibodies in order to multiplex up to 500 targets in a single assay [109,110]. Analytes are quantified by the binding of a biotinylated target-specific detector antibody to a streptavidin-coated fluorescent dye, which then passes through two lasers. The first laser decodes the color-coded bead, while the second quantifies the fluorescence intensity of the associated detector dye. The detection system can be flow based or magnetic based; in the latter, beads are anchored to a specific location by a magnet for imaging. The flow system has a higher multiplexing capability, as immunocomplexes are analyzed individually and sequentially [111]. 

Both the MSD and Luminex immunoassays run into similar pitfalls as other antibody-based techniques, namely, antibody specificity and cross-reactivity, which restrict the number of multiplexable targets. While Luminex boasts the simultaneous measurement of up to 500 analytes, realistically, it is limited to a panel of approximately 30 targets due to antibody cross-reactivity [112]. Although immunoassays are considered high-throughput for sample quantification, the number of multiplexable targets available through these techniques requires the development of a strong hypothesis in order to be used efficiently. Initial biomarker discovery may be more suited to LC-MS/MS strategies, which can then be extended into an ECL immunoassay approach once a select set of proteins of interest has been identified.

### 3.2. Adaptations of Standard Capture Methods

The shortcomings of antibody-based detection techniques have driven the development of new technologies to detect proteins in biofluids. In an attempt to decrease the influence of nonspecific cross-reactivity, OLink proteomics developed the Proximity Extension Assay (PEA) [113,114]. Instead of using one capture and one labeled detection antibody, complementary DNA oligonucleotides are conjugated to both antibodies. The probes only anneal if both antibodies are bound to the same protein. Quantification is performed by qPCR on annealed oligonucleotides, allowing for multiplexing of up to 92 targets with higher sensitivity than a standard ELISA. 

In SOMAscan technology from Somalogic [115], antibodies are entirely replaced by short (20–60 nucleotide) fluorescently labeled DNA Slow Offrate Modified Aptamers (SOMAmers) that can specifically bind over 1100 protein targets. After biotinylation and multiple rounds of washing, aptamers that successfully bind protein targets are bound to a DNA array and quantified by fluorescence intensity. DNA SOMAmers are unlikely to suffer from batch effects as severely as antibodies given they can be easily synthesized, but design and testing of specific probes for thousands of targets requires multiple rounds of optimization and careful quality control procedures. The SOMAscan assay has been shown to have extremely reliable technical reproducibility, with intra- and interplate Coefficients of Variation (CVs) in the ~5% range [19]. As with traditional immunoassays, sources that can introduce variability and contribute to poor (>20%) CVs include dilution factors and proximity to detection limits.

The interpretation of both the Proximity Extension Assay and SOMAscan data is heavily dependent on post-data collection processing algorithms and normalization procedures [19,116]. There are several data treatment methods currently developed for transforming PEA and SOMAscan data, each designed to focus on minimizing a specific source of variability. Differences in data processing can also drastically affect intersite replicability and lead to inconsistencies between reported findings. Standardized data-treatment procedures are necessary in order to ensure concordant interpretation of the data and comparability between study centers.

### 3.3. Ultrasensitive Detection Methods

In a traditional ELISA, sensitivity to lower abundance analytes is reduced due to the dilution of capture-target-detector complexes (immunocomplexes) in a relatively large liquid volume. The limits of detection are therefore related to the optical sensitivity of the detection system. In novel ultrasensitive methods such as single molecule counting (SMC, EMD Millipore) [22] and single molecule array (Simoa, Quanterix) [17], microfluidic technologies spatially isolate immunocomplexes, allowing for significantly more sensitive detection of low-concentration analytes through counting single molecules. In SMC systems, detector antibodies from immunocomplexes are cleaved off to pass through a laser that excites fluorescent tags, allowing each individual detector to be counted as it passes through. Currently, this technology only allows for measurement of a single analyte. In Simoa, intact immunocomplexes are washed into a bead array, where each immunocomplex occupies a single well. This spatial localization allows for detection of a single immunocomplex on each bead, and coupling with different fluorophores allows for multiplexing of up to six analytes. Although SMC and Simoa technology are still antibody-based techniques and maintain similar matrix interference issues to ELISA immunoassays, increased spatial localization allows an algorithm to model the binding of low-abundance antigens, increasing the dynamic range of the system. Analyte concentrations as low as femtogram/mL can now be quantified, as higher dilution factors can be employed without causing analyte concentrations to fall below the detection limits of the assay.

### 3.4. Candidate Disease Markes from Capture-Based Studies

The improved sensitivity and the reduced impact of extreme abundance proteins in capture-based studies in comparison to LC-MS/MS techniques has led to their being used to great effect in blood-derived biofluids. Neurofilament light chain (NfL) may prove to be a useful biomarker of overall neurodegeneration (“N”). In both blood and CSF, NfL is elevated in the presence of neuronal damage, although it is not disease specific [18,117]. Although 50 times more concentrated in CSF than in blood, differences in NfL levels between controls and cognitively impaired individuals are still evident in blood. Although NfL data across various platforms tends to be consistent, the measurements do not always perfectly correlate, and in some cases, significant outcomes are only evident on particular platforms [18]. Such variability between platforms is not peculiar to NfL and has been observed for a number of analytes in multiple studies [111,118]. YKL-40 is another emerging biomarker in Alzheimer’s disease that shows promise in linking neuroinflammation to neurodegeneration. Concentrations of YKL-40 were significantly elevated in CSF (and more modestly increased in plasma) in individuals across various states of dementia [119,120]. However, YKL-40, like NfL, may be reflective of general neuroinflammation and may not necessarily be disease specific. The lack of agreement between different immunoassay technologies can contribute to mixed findings and discrepancies in reported absolute concentrations, complicating the overall understanding of neurodegenerative diseases at a population level. 

Many studies have also proposed panels of various combinations of plasma or serum biomarkers associated with cognitive decline or disease severity that have the potential to profile different aspects of neurodegeneration. Some of the most consistently investigated candidates include proinflammatory cytokine TNF-α, microvascular injury markers ICAM-1 and VCAM-1, and clusterin, an extracellular shuttling protein reported to be associated with Alzheimer’s disease progression [7,121,122,123,124]. Within the literature, there have been discussions regarding conflicting reports of significant associations between proposed markers and disease staging or differential diagnoses [7,124,125], which are attributed to differences in platforms, methods, data processing, and a lack of standardization and reproducibility. Of particular concern is the general under-reporting of nonsignificant analytes in studies that use large-scale multiplexes such as SOMAscan and antibody array-like methods. By only including data of a small subset of analytes (commonly, those that are found to be the most significant) and not making data on the full range of analytes publicly available, it is impossible to tell which of the remaining analytes were confidently detected but not significantly altered with disease. This is an important distinction, as it can inform whether the analyte may still be of interest as opposed to not reliably quantifiable due to limitations of the technology used.

## 4. Considerations for Accurate and Reproducible Findings

If the field wishes to discover reliable, quantifiable biomarkers for neurodegenerative dementias, then data from multiple large studies across heterogeneous populations must be comparable. In the final section of this review, we will discuss some technical considerations important for the accuracy of these techniques and recommendations for reporting that will improve our ability to compare data and achieve sufficient sample sizes to draw population-level conclusions above the variability of human samples.

### 4.1. Preanalytical Effects

In addition to post-data collection processing and platform-specific variability, preanalytical factors can affect the accuracy and reproducibility of measured analytes. The effects of preanalytical factors have already been systematically reviewed [126,127,128]. Here, we aim to emphasize the importance of standardizing these factors to ensure reliable measurements across multiple centers. Preanalytical factors are divided into two subgroups: in vivo and in vitro factors. These factors include but are not limited to: collection methods and materials, hemolytic contamination of samples, sample handling, storage temperature, thaw conditions, sample stability prior to processing, and kit lot-to-lot variability [129,130]. Much has been written on the importance of collecting and storing CSF only with polypropylene plasticware, as polystyrene or other materials can bind very sticky proteins such as amyloid-β or prion proteins [131,132]. Freeze-thaw cycles (the number of times a stored sample is thawed and refrozen) are often investigated as a cause of protein degradation over repeated uses [133]. Protein integrity varies across analytes and biofluids and maximum acceptable freeze-thaw cycles are specific to each platform, depending on detection sensitivity. Ideally, sample collection methods and times should be strictly controlled to minimize diurnal effects, as well as accounting for possible differences in analyte concentrations between fasting and nonfasting biofluids, which can affect levels of hormones, triglycerides, and other metabolic-pathway-related markers. Levels of certain proteins may vary widely day to day, and thus it is also important to examine the biotemporal stability of an analyte before considering its use as a biomarker [23].

### 4.2. Matrix Effects

Biofluid composition is also an important consideration when using a multiplex immunoassay system. Matrix effects can negatively impact the ability of highly sensitive immunoassays to accurately quantify certain analytes [134]. As with label-free proteomics techniques, complex matrices with high abundance of albumin and immunoglobulins can affect antibody binding and increase background, masking low-abundance proteins. These low-abundance proteins often approach immunoassay limits of detection, increasing the difficulty of accurate quantification. In some cases, such as with CSF, increasing sample volume may allow for the detection of these low abundance proteins. However, for more complex biofluids, the sample matrix has been found to inhibit detection of certain analytes in spike-recovery experiments, and increasing sample volume would not improve quantification [135]. In a comparison between standards of known concentrations spiked in immunoassay buffer versus serum and plasma matrices, analyte quantification was significantly lower in the presence of either human sample matrix compared to the buffer. This inhibitory effect has been investigated by a number of other studies researching the quantification of low-abundance proteins in complex biofluids [136,137]. 

These sources of interference in immunoassay detection can lead to misinterpretation of assay results, which can affect clinical or research outcomes. Inhibitory effects may vary between immunoassay detection systems and contribute to inaccurate measurements, increasing the difficulty of comparing quantification across multiple platforms. Due to possible matrix effects, it is generally recommended that the interpretation of analyte quantification in undiluted samples be relative rather than absolute; that is, the measurement should be interpreted in relation to other sample concentrations measured using the same platform. Dilution of samples in immunoassay buffers often improves quantification accuracy by mitigating such matrix effects, resulting in more absolute quantification. When investigating a new immunoassay, it is important to take into consideration possible sources of interference and assess dilution linearity and spike-recovery performance to determine optimal sample conditions. Some assays may not be suited to analyte detection in all matrices, as each sample matrix requires individual optimization. For CSF, dilution factors may be necessary for absolute quantification but can cause analyte measurements to fall below the limit of detection.

### 4.3. Data Processing

The difficult challenge of how to standardize data comes from the technical aspects of the proteomic workflow. The adoption of different quantification techniques for proteins of variable abundance makes comparison across studies difficult. LC performance can vary substantially over time and can introduce significant variability to an experiment [36]. Simple measures can be taken to improve monitoring of day-to-day instrument variability and demonstrate instrument reliability, such as spiking with retention time calibrators and monitoring of abundant peptides in automatic QC systems like AutoQC in Panorama [138]. 

How to appropriately normalize data and compare across studies is a more difficult problem with very little consensus, and the field should consider a series of questions. The first regards whether input protein concentration should be normalized before proteomic quantification, as is standard in LC-MS/MS workflows, or whether the same volume of each fluid should be used per assay (as applies to ELISA workflows). The second is whether distribution-based normalization methods (e.g., median or quantile normalization) are appropriate in this context, given that they are based on the assumption that most analytes will not change in abundance between conditions, and that a roughly symmetric proportion of proteins will increase and decrease in abundance. If the integrity of the blood brain barrier is compromised by a neurodegenerative process, this may lead to proteome-wide increases in CSF protein concentration, invalidating the assumption that most proteins will not change in abundance between conditions [139]. Where panels of proteins have been selected on the basis that they are likely to vary between disease conditions, the same assumption is also invalidated and distribution-based normalization may be rendered inappropriate. The alternative approach, to select a subset of “housekeeping” proteins to which to normalize, is also problematic, as a number of studies have shown significant disease-related differences in the abundant biofluid proteins, which would be the most obvious candidates for selection. We would argue that there is currently insufficient high-quality data available to select a panel of normalizing peptides/proteins that may be stable across neurodegenerative conditions, and establishing whether such stable proteins exist should be an additional priority of hypothesis-free proteomic experiments. The current gold standard in quantification and reproducibility, therefore, may be smaller-scale targeted experiments, where ratiometric comparisons to a heavy-labeled standard with proven linearity or a standard curve allowing reporting of a concentration may be the most reliable means of quantification. As this approach does not allow for hypothesis-free discovery, these approaches should be used in replication cohorts for findings that arise from untargeted methods.

### 4.4. Multisite Variability

It is important to conduct replication studies to assess intersite and interuser variability using the same platform and data-processing methods. Seemingly trivial or unapparent differences in techniques, materials, or environmental conditions can affect results. It is not sufficient to assume that employing the same sample-processing procedures, the same multiplex assay kits or LC setup, and standardized data reporting will necessarily eliminate variability. In an extensive multisite study involving six different labs, Breen et al. [118] found that each analyte measured showed at least one significant lab or assay lot-to-lot effect despite following a consensus protocol across all sites. Care should be taken to establish systems of determining assay reproducibility, such as including standardized plate-to-plate controls to minimize plate effects across multiple sites and batch ordering assays to ensure lot consistency. Even so, controlling for every source of variability and assessing the performance of all available technologies and platforms is often unrealistic due to financial and resource limitations.

## 5. Future Directions

Proteomics is a relatively new and rapidly growing field and has yet to develop clear standards for reporting data and consistent methods to allow for confident comparison of datasets. The complexity of and similarity between neurodegenerative diseases means that studies of large, diverse populations are required to define biomarkers that are both sensitive and specific. It is therefore of critical importance that the field as a whole adopts stringent and detailed reporting criteria to build knowledge on a scale that will help delineate and stratify subjects across populations in a biologically informative manner. While proteomic-specific journals have begun to adopt set reporting criteria, clinical journals do not generally require this level of detail, and the field suffers as a result. At a bare minimum, a data table that includes every peptide and/or protein confidently detected in each proteomic experiment (including retention time and mz data for LC-MS/MS), abundance in each individual sample, and per group summary statistics should be provided for every study. A list of significantly changed proteins with a fold change and p/q value is not sufficient for thorough examination of the data. As a field, a decision should be made to use a standardized protein reference, as switching between Uniprot IDs [140], gene names, and other reference formats often leads to errors and data loss. We propose the use of both the Ensembl gene ID [141], which is clearly linked to genomic locus and reference version, and a more descriptive gene ID such as the gene symbol for ease of understanding results. Similarly, clinical and demographic data should be provided on an individual subject level to allow for modeling of age, sex, and other important demographic variables. The development and adoption of user-friendly resources such as the CSF Proteome Resource and Plasma Proteome Database [14,142] to allow for cross-study comparison is also critically important. Adoption of standards along these lines will likely lead to leaps forward in the biomarker discovery pipeline equivalent to the speed at which the discovery technology is improving.

## Figures and Tables

**Table 1:** Summary table showing cross-study results from the proteins illustrated in Figure 2. The arrow shows the direction of change in the neurodegenerative disease compared to controls. PD: Parkinson’s Disease, LBD: Lewy Body Dementia, APS: Atypical Parkinsonism, FTD: Frontotemporal Dementia.

Protein	Gene Symbol	Mild Cognitive Impairment	Alzheimer’s Disease	Amyotrophic Lateral Sclerosis	Other Diseases
Serum albumin	ALB	↔ [89,90]	↓ [48,91,92] ↑ [48,92] ↔ [89,90]	↔ [93,94,95]	
Amyloid Beta Precursor Like Protein	APLP1	↑ [96] ↔ [89,90]	↔ [89,90,96,97,98] ↓ [91] ↑ [98]	↔ [94,95]	↓ PD [98]
Apolipoprotein E	APOE	↓ [89] ↔ [90]	↑ [48,92,99,100] ↔ [83,90,91,97] ↓ [89]	↔ [93,94,95]	↔ PD [98,99] ↑ LBD [99]
Amyloid Precursor Protein	APP	↔ [90]	↔ [83,89,90,96] ↓ [97]	↔ [93,94,95]	↔ PD [98,99] ↑ LBD [99] ↓ APS [101]
Chromogranin A	CHGA	↔ [89,90]	↓ [91,97,102] ↔ [89,90,92]	↔ [93,94,95]	
Chitinase 3 Like 1 (YKL-40)	CHI3L	↔ [89,90]	↑ [90,99,100] ↔ [83,89]	↔ [93,94] ↑ [95]	↔ PD [99] ↑ LBD [99] ↑ FTD [103] ↑ APS [101]
Cystatin-C	CST3	↔ [89,90]	↓ [102] ↑ [92,99,100] ↔ [89,90,91,97]	↔ [93,95] ↓ [94]	↔ PD [98,99] ↑ LBD [99]
Insulin Like Growth Factor-2	IGF2	↔ [89]	↑ [99,100] ↔ [89]	↓ [93] ↔ [95]	↔ PD [99] ↑ LBD [99]
Neuronal Pentraxin 1	NPTX1	↓ [89] ↔ [96]	↓ [89,102] ↔ [83,96]	↔ [93,94,95]	↔ PD [98] ↓ APS [101]
Secretogranin-2	SCG2	↔ [96]	↓ [91,102] ↔ [83,96]	↔ [93,95] ↓ [94]	↓ APS [101]
Secretogranin-3	SCG3	↔ [89,96]	↔ [83,89,96] ↓ [91,97] ↑ [48]	↔ [93,94,95]	↓ APS [101]
Transthyretin	TTR	↑ [89,90]	↑ [90,92,99] ↔ [83,91,97,100]	↔ [93,94]	↔ PD [99] ↔ LBD [99]
Ubiquitin (mono/poly)	UBB		↑ [48,99,104,105] ↔ [83]	↔ [94,95,104]	↔ FTD [104] ↔ APS [105] ↑ LBD [99] ↔ PD [99,104,105]
Neurosecretory Protein VGF	VGF	↔ [89,96]	↓ [91,97,102] ↔ [83,89,96]	↔ [93,94,95]	↓ APS [101]
